# Change in the hormone receptor status following administration of neoadjuvant chemotherapy and its impact on the long-term outcome in patients with primary breast cancer

**DOI:** 10.1038/sj.bjc.6605360

**Published:** 2009-10-06

**Authors:** T Hirata, C Shimizu, K Yonemori, A Hirakawa, T Kouno, K Tamura, M Ando, N Katsumata, Y Fujiwara

**Affiliations:** 1 Department of Breast and Medical Oncology, National Cancer Center Hospital, Chuo-ku, Tokyo 104-0045, Japan; 2 Department of Management Science, Graduate School of Engineering, Tokyo University of Science, Shinjuku-ku, Tokyo 162-8601, Japan

**Keywords:** breast cancer, endocrine therapy, hormone receptor status change, neoadjuvant chemotherapy, prognosis

## Abstract

**Background:**

To evaluate the impact of change in the hormone receptor (HR) status (HR status conversion) on the long-term outcomes of breast cancer patients treated with neoadjuvant chemotherapy (NAC).

**Methods:**

We investigated 368 patients for the HR status of their lesions before and after NAC. On the basis of the HR status and the use/non-use of endocrine therapy (ET), the patients were categorised into four groups: Group A, 184 ET-administered patients with HR-positive both before and after NAC; Group B, 47 ET-administered patients with HR status conversion; Group C, 12 ET-naive patients with HR status conversion; Group D, 125 patients with HR-negative both before and after NAC.

**Results:**

Disease-free survival in Group B was similar to that in Group A (hazard ratio, 1.16; *P*=0.652), but that in Group C was significantly lesser than that in Group A (hazard ratio, 6.88; *P*<0.001). A similar pattern of results was obtained for overall survival.

**Conclusion:**

Our results indicate that the HR status of tumours is a predictive factor for disease-free and overall survival and that ET appears to be suitable for patients with HR status conversion. Therefore, both the CNB and surgical specimens should be monitored for HR status.

Neoadjuvant chemotherapy (NAC) was introduced in the early 1980s for patients with locally advanced breast cancer, initially to improve the operability of tumours (Kaufmann *et al*, 2003). Recently, the application of this therapy has been extended to cases of operable disease. The previously reported results of a meta-analysis indicated that neoadjuvant and adjuvant chemotherapy are equivalent in terms of overall survival (OS) and disease-free survival (DFS) (Mauri *et al*, 2005). As the pertinent published reports present conflicting views, the actual indications for NAC remain controversial.

Before the initiation of NAC, core-needle biopsy (CNB) is often performed to establish the histological diagnosis and to assess certain factors considered predictive of treatment outcomes. The hormone receptor (HR) status is one such factor. Although this status is known to change after NAC (Bottini *et al*, 1996; Lee *et al*, 2003; Taucher *et al*, 2003; Colleoni *et al*, 2004; Burcombe *et al*, 2005; Shet *et al*, 2007; Tacca *et al*, 2007; Kasami *et al*, 2008; Neubauer *et al*, 2008), its impact on long-term outcomes has not been assessed. The objective of this retrospective study was to evaluate the frequency and impact of change in the HR status (HR status conversion) on the long-term outcomes in the NAC-administered breast cancer patients.

## Materials and methods

### Patients

We selected 459 primary breast cancer patients treated at the National Cancer Center Hospital between May 1995 and July 2007. All the patients had received anthracycline- and taxane-based NAC. The clinical stages of the patients ranged from cT2N0M0 to cT4dN3M0, which includes inflammatory (T4d) carcinoma. Data were collected on the pre- and post-NAC statuses of oestrogen receptor (ER), progesterone receptor (PgR), and human epidermal receptor (HER) 2 expressions in the lesions. Patients in whom pathologic complete response (pCR) was obtained (91 patients) after surgery, including those with only residual ductal carcinoma *in situ* (DCIS), were excluded from this analysis because the HR status of the lesions of these patients could not be accurately evaluated. The remaining 368 patients were classified into four groups on the basis of the HR status of their lesions before and after NAC and the use/non-use of endocrine therapy (ET): Group A, 184 ET-administered patients with lesions that were HR-positive both before and after NAC; Group B, 47 ET-administered patients with lesions showing HR status conversion; Group C, 12 ET-naive patients with lesions showing HR status conversion; Group D, 125 patients with lesions that were HR-negative both before and after NAC. The mean age at the time of diagnosis of breast cancer was almost the same in the four groups.

### Hormone status and HER2 status determination

All the patients underwent CNB performed using an 18G needle. The ER, PgR, and HER2 statuses of all the CNB and surgical specimens were determined by immunohistochemistry (IHC). Details regarding the antibodies used, the clones used, and the time periods for which they were used, as well as the antigen retrieval and the source of antibodies for IHC studies, are listed in Table 1. Positive staining for ER/PgR was defined as nuclear staining in ⩾10% of the tumour cells. HER2 protein overexpression was defined as with 3+ complete membrane staining. If HER2 staining on IHC was determined to be 2+, fluorescent *in situ* hybridisation (FISH) was used to confirm the results. FISH was performed using the PathVysion kit (Abott-Vysis Lab, Abott Park, IL, USA). HER2 gene amplification was defined as a HER2:chromosome 17 ratio of ⩾2.1. HR positivity was defined as positivity for ER and/or PgR. The Allred scoring system was used to assess the degree of staining (Allred *et al*, 1998). Standard controls were prepared on a daily basis for each tumour to ensure the results of IHC.

### Tumour size determination and evaluation of neoadjuvant chemotherapy response

Before each chemotherapy treatment and before surgery, the two greatest perpendicular diameters of the tumours in the breast and axillary nodes were measured, and the products of these diameters were added as a measure of total tumour size. No clinical response of palpable tumour in the breast and axillary lymph nodes was defined as a complete response (CR). Reduction in total tumour size of 50% or greater was graded as a partial response (PR). An increase in total tumour size of more than 50% or the appearance of new suspicious ipsilateral axillary adenopathy was considered as a progressive disease (PD). Tumours that did not meet the criteria for objective response or progression were considered as a stable disease (SD).

### Chemotherapy

Patients receiving NAC were administered an anthracycline and a taxane, either concurrently or sequentially. Those receiving concurrent therapy were administered four cycles (doxorubicin at 50 mg m^−2^ plus docetaxel at 60 mg m^−2^) every 21 days. Patients showing clinical CR or PR to the above treatment were administered two additional cycles of the same regimen after the surgery. However, patients who did not achieve objective clinical response to NAC were administered with four cycles of 5-fluorouracil (600 mg m^−2^), methotrexate (40 mg m^−2^), and cyclophosphamide (600 mg m^−2^) after the surgery. For patients receiving the sequential regimen, four cycles of 5-fluorouracil (500 mg m^−2^), epirubicin (100 mg m^−2^), cyclophosphamide (500 mg m^−2^) or doxorubicin (60 mg m^−2^), and cyclophosphamide (600 mg m^−2^) were administered every 21 days, followed by a taxane. As a taxane, paclitaxel was administered weekly at a dose of 80 mg m^−2^ per week for 12 weeks or at a dose of 175 mg m^−2^ every 3 weeks for four cycles, or docetaxel was administered every 3 weeks at a dose of 75 mg m^−2^ for four cycles.

### Adjuvant endocrine therapy (ET) and irradiation

Adjuvant radiotherapy was administered to patients who underwent breast-conserving surgery. Adjuvant radiotherapy was recommended to those who underwent modified radical mastectomy for the disease ranging from cT3N1M0 to cT4dN3M0. The decision to administer ET was taken on the basis of the treating physician's and/or the patient's preferences. Most patients with HR-positive lesions were administered 20 mg of tamoxifen daily for 5 years. From 2005 onwards, postmenopausal women taking tamoxifen were (1) allowed to switch to an aromatase inhibitor before completing 5 years of tamoxifen, (2) allowed to begin taking an aromatase inhibitor after a 5-year course of tamoxifen or (3) recommended an aromatase inhibitor for the first 5 years.

### Statistical analysis

The frequencies and descriptive statistics of the demographic and clinical variables from the four groups—A, B, C, and D—were obtained. The ER and PgR statuses of the lesions before and after NAC were compared using the consistency test. DFS was defined as the time from surgery to the detection of relapse, death from any cause, or the date of the last visit for patients without events. OS was defined as the time from surgery to death from any cause or the date of the last visit for patients without events. DFS and OS were estimated using the Kaplan–Meier method, and the survival curves were compared using the log-rank test. Multivariate Cox regression analysis with stepwise selection (*α*=0.05) was used to estimate the hazard ratio, 95% confidence interval (CI), and the effects of the clinical and pathological variables. A two-sided *P*<0.05 was considered to be statistically significant. All the analyses were performed using the SAS (version 9.1; SAS Institute Inc., Cary, NC, USA).

## Results

### Patient characteristics

Among the 459 NAC-administered patients, pCR after surgery was achieved in 91 patients. Pathological assessment of the CNB specimens of patients with pCR revealed that 26 (28.6%) and 19 (20.9%) patients, respectively, were ER-positive and PgR-positive and that 63 (69.2%) were negative for both ER and PgR.

Examination of the surgical specimens revealed residual invasive disease in 368 patients. The distribution of these patients in the four groups was as follows: Group A, 184 (50.0%) patients; Group B, 47 (12.8%) patients; Group C, 12 (3.3%) patients; Group D, 125 (34.0%) patients.

The patient and tumour characteristics of the four groups are listed in Table 2. The postoperative performance status (PS 0 or 1) of all the patients was good. HR status conversion after NAC was observed in 59 (16.0%) patients. None of the HER2-positive patients were administered trastuzumab during neoadjuvant or adjuvant chemotherapy. Twelve (3.3%) did not receive adjuvant ET, although it was not contraindicated, but all of their lesions showed HR status conversion after NAC. All patients whose lesions showed ER status from positive to negative after chemotherapy had been administered ET.

### Change in the HR status and HER2 status

**Figure 1:**
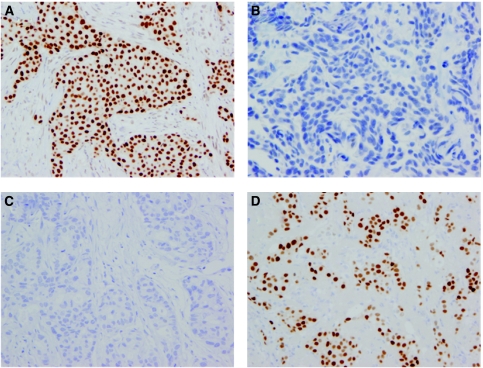
Immunostaining for oestrogen receptor in core needle biopsy and surgery specimens after neoadjuvant chemotherapy. (**A**) Staining of tumour cells in core-needle biopsy sample (CNB) staining positively for oestrogen receptor (ER). (**B**) Staining of tumour cells in surgical samples with ER-negative status after neoadjuvant chemotherapy (NAC). (**C**) Staining of tumour cells in CNB specimens with ER-negative status. (**D**) Staining of tumour cells in surgical samples with ER-positive status after NAC.

The typical staining patterns of the CNB and surgical specimens are shown in Figure 1. The pre- and post-NAC ER and PgR statuses are shown in Table 3. Lesions of 23 (6.3%) patients showed a change in both the ER and PgR statuses after NAC. The HR and HER2 statuses changed from positive to negative in 30 (8.2%) and 22 (6.0%) patients, and changed from negative to positive in 29 (7.9%) and 13 (3.5%) patients, respectively.

**Figure 2:**
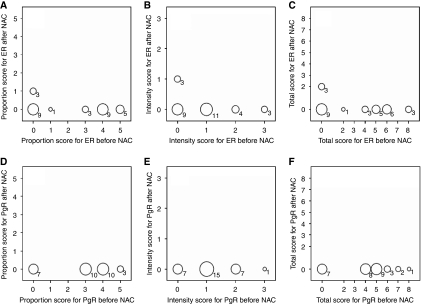
The distribution of scores for oestrogen receptor and progesterone receptor staining before and after neoadjuvant chemotherapy in 30 patients whose lesions changed from hormone receptor (HR)-positive status to HR-negative status. The size of the circle indicates the number of patients and the number is below the circle. (**A**–**C**) Proportion score, intensity score and total score of ER before and after NAC. (**D**–**F**) Proportion score, intensity score and total score of PgR befoe and after NAC.

**Figure 3:**
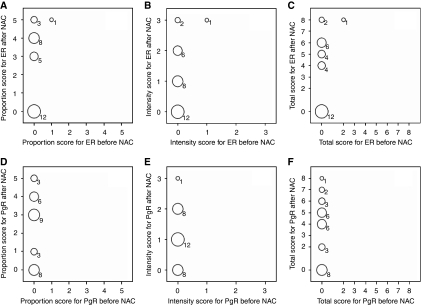
Scores of staining for oestrogen receptor and progesterone receptor before and after neoadjuvant chemotherapy in 29 patients whose lesions changed from being hormone receptor (HR)-negative to HR-positive. The size of the circle indicates the number of patients and the number is below the circle. (**A**–**C**) Proportion score, intensity score, total score of ER before and after NAC. (**D**–**F**) Proportion score, intensity score, total score of PgR before and after NAC.

**Figure 4:**
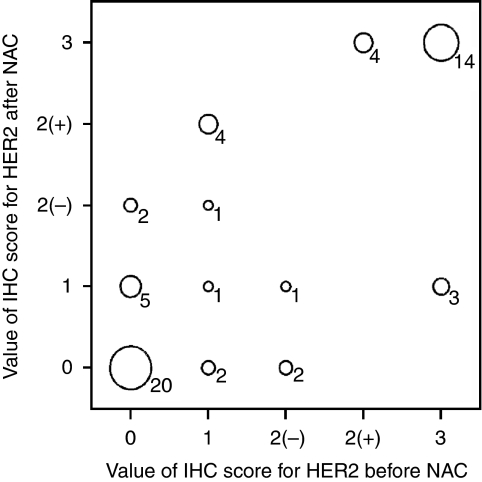
Bubble plot for immunohistochemistry score for HER2 before and after neoadjuvant chemotherapy in 59 patients with hormone receptor status conversion. The figures added to the bubbles are the number of patients and each bubble's size is determined by the number of patients in the category: the more the patients, the larger the bubble. The symbols (+) and (−), respectively, indicate the positive and negative status by fluorescent *in situ* hybridisation (FISH).

Figures 2 and 3 show the pre- and post-NAC proportion, intensity, and total scores of ER and PgR staining, determined on the basis of the Allred scoring system, for patients who underwent a change in the HR status. As shown in Figures 2 and 3, the changes in the ER and PgR statuses were observed not only in cases with borderline positive (total score 3–5) staining but also in those with strongly positive (total score 7–8) staining. The change in the HR status was not caused by a change in only the proportion or intensity scores of ER and PgR. In addition, Figure 4 shows the results of HER2 testing in the 59 patients who showed HR status conversion.

### Long-term outcomes

**Figure 5:**
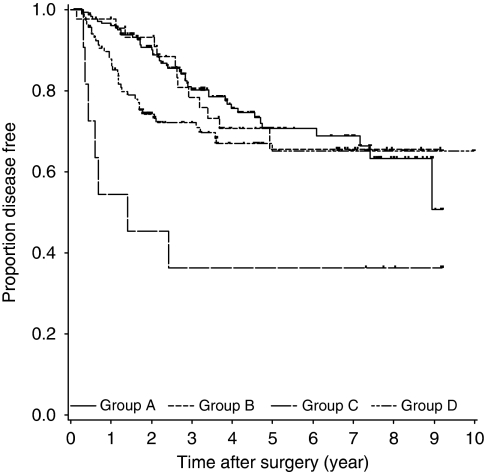
Kaplan–Meier curves of disease-free survival in four groups. Short vertical lines indicate censored data points. Log-rank test was significant for disease-free survival (DFS) (*P*=0.008).

The median duration of follow-up was 47 months. Figure 5 shows the Kaplan–Meier curves for DFS in the four groups. The differences among the four curves were statistically significant, as determined by the log-rank test (*P*=0.008). The 3-year DFS rates in Groups A, B, C, and D were 80.3, 78.4, 36.4, and 72.2%, respectively.

Table 4 shows the results of the multivariate Cox regression analysis of DFS with stepwise selection. The following six variables were chosen as prognostic factors for inclusion in the Cox proportional hazard model: age (<35 *vs* ⩾35 years), clinical stage at diagnosis (IIA and IIB, or IIIA *vs* IIIB or IIIC), histological grade (1 *vs* 2 and 3), HER2 status (positive *vs* negative), clinical response (CR, PR *vs* SD, PD), and the number of lymph node metastases (0 *vs* 1–3 *vs* ⩾4). Three of these variables—the HER2 status, clinical response to NAC, and the number of lymph node metastases—were identified by the stepwise selection method in the multivariate Cox regression model as the variables affecting the DFS.

The DFS of Groups B and A was similar (hazard ratio, 1.16; 95% CI, 0.61–2.19), whereas that of Group C was significantly shorter than that of Group A (hazard ratio, 6.88; 95% CI, 3.00–15.80). Table 5 summarises the results of the analysis of the efficacy of ET in the 59 patients who showed HR status conversion by using the multivariate Cox regression model. The DFS of the ET-administered patients was significantly longer than that of ET-naive patients (hazard ratio, 0.19; 95% CI, 0.06–0.60; *P*<0.004).

**Figure 6:**
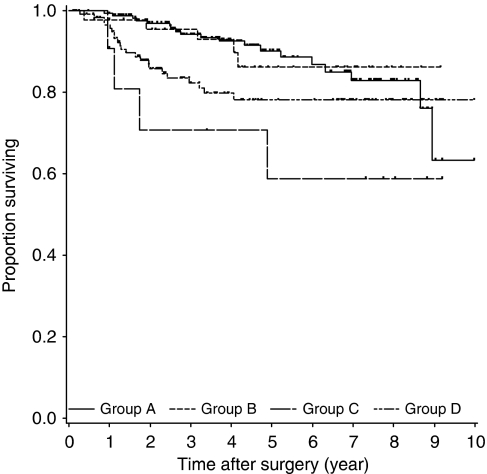
Kaplan–Meier curves of OS in four groups. Short vertical lines indicate censored data points. Log-rank test was significant for overall survival (OS) (*P*=0.035).

Figure 6 shows the Kaplan–Meier curves for OS in the four groups. The differences among the four curves were statistically significant, as determined by the log-rank test (*P*=0.035). The 5-year survival rates of Groups A, B, C, and D were 90.3, 86.3, 58.9, and 78.2%, respectively. The pattern of results of the analyses for OS in the four groups was similar to that for DFS.

## Discussion

This is the first report on the long-term outcomes and impact of adjuvant ET in patients with HR status conversion after NAC. In this study, the DFS and the OS of ET-administered patients with HR status-converted lesions were similar to those of ET-administered patients with lesions that were HR-positive both before and after NAC, whereas the DFS of ET-naive patients whose lesions show HR status conversion was significantly shorter than that of ET-administered patients whose lesions were HR-positive both before and after NAC. Analysis of OS yielded results similar to that pertaining to DFS. These findings indicate that the change in the status alone did not seem to influence the long-term outcome; rather, the non-administration of adjuvant ET seemed to be associated with a worse prognosis.

ER, PgR, and HER2 status changes were observed in 14.9, 29.1, and 9.5% of the patients included in our study. The overall frequency of patients with HR status conversion was 16.0%. This incidence of HR status conversion was similar to previous reports on post-NAC change in the ER, PgR, and HER2 statuses, which reported incidences of 8–28%, 6–59% (Bottini *et al*, 1996; Lee *et al*, 2003; Taucher *et al*, 2003; Colleoni *et al*, 2004; Burcombe *et al*, 2005; Shet *et al*, 2007; Kasami *et al*, 2008; Neubauer *et al*, 2008), and 0–21% (Bottini *et al*, 1996; Colleoni *et al*, 2004; Arens *et al*, 2005; Burcombe *et al*, 2005; Quddus *et al*, 2005; Adams *et al*, 2008; Kasami *et al*, 2008; Neubauer *et al*, 2008), respectively. Although the rate of cases with no change in the HR status after NAC was high, the incidence of change in the HR status is clinically not negligible. The poor prognosis of patients with HR status conversion not administered adjuvant ET indicates the necessity to determine the HR status of the lesions both before and after NAC and to administer ET to patients with HR status conversion.

Despite yielding these clinically relevant findings, our study is limited in some aspects: (1) The patient groups studied were heterogeneous in terms of sample size and characteristics. (2) This study was retrospective and the results of the statistical tests were not based on randomisation, but were exploratory, although the prognostic factors were adjusted using multivariate Cox regression analysis. Therefore, the impact of the change in the pre- and post-NAC HR statuses on the long-term outcomes and the efficacy of ET for patients with HR status conversion should be evaluated using a prospective study design. (3) The methods for measuring the ER and PgR status varied with the age of the patients, as shown in Table 1. Although the methods used for the determination of the HR statuses of the tumours of 36 patients among 368 patients were measured using different methods for the CNB and surgical specimens, only three of these tumours showed HR status conversion. A previous report showed that the HR status conversion occurred in 23% of the population in a study in which the same methods were used for the analysis of CNB and surgical specimens (Tacca *et al*, 2007), whereas HR status conversion was observed in 16.0% (59 patients) of the patients in this study. Therefore, the difference in the methods for measuring the ER and PgR statuses of the CNB and surgical specimens seems not to be the only reason for HR-status conversion.

In conclusion, our study showed that the prognosis of patients with change in HR status after NAC but who did not receive ET was worse than that of the other groups. The hormone receptor status should be evaluated not only in the biopsy specimens obtained before the initiation of NAC but also in specimens obtained during post-NAC surgery; the pre- and post-NAC HR statuses will help determine the indication for adjuvant ET in patients. ET appears to be suitable for patients with tumours positive for HR status at least once, that is, either before or after NAC.

## Figures and Tables

**Table 1:** Panel of antibodies

**Antigen**	**Period used for**	**Clone**	**Type**	**Antigen retrieval**	**Source**
ER	Until Oct 2002	1D5	Mouse monoclonal	A/C citrate buffer	Dako
	From Nov 2002 to Feb 2005	ER88	Mouse monoclonal	As above	Bio Genex
	From Mar 2005	1D5	Mouse monoclonal	As above	Dako
PgR	Until Oct 2002	1A6	Mouse monoclonal	As above	Novocastra
	From Nov 2002 to Feb 2005	PR88	Mouse monoclonal	As above	Bio Genex
	From Mar 2005	PgR636	Mouse monoclonal	As above	Dako
HER2	Until Oct 2002	c-erbB-2	Rabbit polyclonal	As above	Dako
	From Nov 2002 to Feb 2005	CB11	Mouse monoclonal	As above	Bio Genex
	From Mar 2005	c-erbB-2	Rabbit polyclonal	As above	Dako

Abbreviations: A/C: autoclave for 10 min at 121°C; ER: estrogen receptor; HER2: human epidermal receptor 2; PgR: progesterone receptor; citrate buffer: 10 mM citrate buffer, pH 6.0.

**Table 2:** Patient and tumour characteristics

**Characteristics**	**Group A (*N*=184)**	**Group B (*N*=47)**	**Group C (*N*=12)**	**Group D (*N*=125)**
Mean±StdDev age, years	48.7±9.9	49.0±9.5	49.5±8.1	49.7±8.8
				
*Tumour stage*				
T1	1 (0.5)	0 (0.0)	1 (8.3)	1 (0.8)
T2	99 (53.8)	20 (42.6)	4 (33.3)	52 (41.6)
T3	46 (25.0)	19 (40.4)	5 (41.7)	50 (40.0)
T4a–c	36 (19.6)	6 (12.8)	2 (16.7)	20 (16.0)
T4d	2 (1.1)	2 (4.3)	0 (0.0)	2 (1.6)
				
*N stage*				
N0	95 (51.6)	22 (46.8)	5 (41.7)	55 (44.0)
N1	67 (36.4)	21 (44.7)	6 (50.0)	51 (40.8)
N2	16 (8.7)	4 (8.5)	1 (8.3)	15 (12.0)
N3	6 (3.3)	0 (0.0)	0 (0.0)	4 (3.2)
				
*Clinical stage*				
IIA	59 (32.1)	12 (25.5)	2 (16.7)	26 (20.8)
IIB	49 (26.6)	15 (31.9)	5 (41.7)	39 (31.2)
IIIA	35 (19.0)	12 (25.5)	3 (25.0)	34 (27.2)
IIIB	36 (19.6)	8 (17.0)	2 (16.7)	22 (17.6)
IIIC	5 (2.7)	0 (0.0)	0 (0.0)	4 (3.2)
				
*Histological grade*				
G1	14 (7.61)	3 (6.4)	1 (8.3)	2 (1.6)
G2	111 (60.3)	26 (55.3)	4 (33.3)	40 (32.0)
G3	57 (31.0)	16 (34.0)	7 (58.3)	78 (62.4)
Unknown	2 (1.1)	2 (4.3)	0 (0.0)	5 (4.0)
				
*HR status before NAC*				
Positive	184 (100.0)	29 (61.7)	1 (8.3)	0 (0.0)
Negative	0 (0.0)	18 (38.3)	11 (91.7)	125 (100.0)
				
*HER2 status before NAC*				
Positive	34 (18.5)	17 (36.2)	4 (33.3)	57 (45.6)
Negative	150 (81.5)	30 (63.8)	8 (66.7)	68 (54.4)
				
*HR status after NAC*				
Positive	184 (100.0)	18 (38.3)	11 (91.7)	0 (0.0)
Negative	0 (0.0)	29 (61.7)	1 (8.3)	125 (100.0)
				
*HER2 status after NAC*				
Positive	26 (14.1)	18 (38.3)	4 (33.3)	55 (44.0)
Negative	158 (85.9)	29 (61.7)	8 (66.7)	70 (56.0)
				
*NAC regimen*				
AT	69 (37.5)	22 (46.8)	10 (83.3)	62 (49.6)
AC followed by T	56 (30.4)	9 (19.2)	2 (16.7)	24 (19.2)
CEF followed by T	59 (32.1)	16 (34.0)	0 (0.0)	39 (31.2)
				
*Clinical response*				
CR/PR	157 (85.3)	40 (85.1)	12 (100.0)	103 (82.4)
SD/PD	27 (14.7)	7 (14.9)	0 (0.0)	22 (17.6)
				
*Operation*				
Lumpectomy	65 (35.3)	18 (38.3)	3 (25.0)	40 (32.0)
Mastectomy	119 (64.7)	29 (61.7)	9 (75.0)	85 (68.0)
				
*Radiotherapy*				
Yes	127 (69.0)	30 (63.8)	9 (75.0)	82 (65.6)
No	57 (31.0)	17 (36.2)	3 (25.0)	43 (34.4)
				
*Number of lymph node metastases*				
0	65 (35.3)	22 (46.8)	3 (25.0)	58 (46.4)
1–3	59 (32.1)	13 (27.7)	5 (41.7)	42 (33.6)
4>	60 (32.6)	12 (25.5)	4 (33.3)	25 (20.0)
				
*Endocrine therapy*				
TAM, 5 years	112 (60.9)	30 (63.8)	0 (0.0)	0 (0.0)
TAM followed by AI	44 (23.9)	11 (23.4)	0 (0.0)	0 (0.0)
AI, 5 years	28 (15.2)	6 (12.8)	0 (0.0)	0 (0.0)
None	0 (0.0)	0 (0.0)	12 (100.0)	125 (100.0)

Abbreviations: AC=doxorubicin and cyclophosphamide; AI=aromatase inhibitor; AT=doxorubicin and docetaxel; CEF=cyclophosphamide, epirubicin and 5-fluorouracil; ER=estrogen receptor; HER2=human epidermal receptor; *N*=number of patients; PgR=progesterone receptor; T=taxane (weekly or triweekly paclitaxel, or triweekly docetaxel); TAM=tamoxifen;

Figures in parentheses are percentage of patients except for age.

**Table 3:** Number of patients classified by estrogen receptor and progesterone receptor statuses before and after neoadjuvant chemotherapy

	**(ER, PgR) after NAC**			
**(ER, PgR) before NAC**	**(+, +)**	**(+, −)**	**(−, +)**	**(−, −)**
(+, +)	69 (18.8)	41 (11.1)	3 (0.8)	10 (2.7)
(+, −)	18 (4.9)	27 (7.3)	1 (0.3)	7 (1.9)
(−, +)	11 (3.0)	6 (1.6)	8 (2.2)	13 (3.5)
(−, −)	6 (1.6)	11 (3.0)	12 (3.3)	125 (34.0)

Abbreviations: ER=estrogen receptor; HER2=human epidermal receptor 2; PgR=progesterone receptor.

Figures in parentheses are percentage of patients.

**Table 4:** Results of multivariate Cox regression analysis of disease-free survival with stepwise selection

**Variables**	**Hazard ratio (95% CI)**	***P*-value**
*Group*		
A	1	
B	1.16 (0.61, 2.19)	0.652
C	6.88 (3.00, 15.80)	<0.001
D	1.63 (1.01, 2.63)	0.045
		
*Clinical stage*		
IIA/IIB/IIIA	1	
IIIB/IIIC	1.56 (1.00, 2.42)	0.049
		
*HER2*		
Negative	1	
Positive	2.00 (1.30, 3.09)	0.002
		
*Clinical response*		
SD/PD	1	
PR/CR	0.56 (0.34, 0.92)	0.021
		
*Number of lymph node metastases*		
0	1	
1–3	2.09 (1.14, 3.83)	0.017
>4	6.49 (3.71, 11.37)	<0.001

Abbreviations: CI=confidence interval; CR=complete response; PR=partial response; SD=stable disease; PD=progression disease.

**Table 5:** Efficacy of endocrine therapy in patients with lesions showing hormone receptor status conversion after neoadjuvant chemotherapy in terms of disease-free survival

**Variables**	**Hazard ratio (95% CI)**	***P*-value**
*ET*		
No	1	
Yes	0.19 (0.06, 0.60)	0.004
		
*HER2*		
Negative	1	
Positive	1.58 (0.46, 5.42)	0.467
		
*Clinical response*		
SD/PD	1	
PR/CR	0.75 (0.15, 3.93)	0.738
		
*Clinical stage*		
IIA/IIB/IIIA	1	
IIIB/IIIC	1.03 (0.26, 4.16)	0.968
		
*Number of lymph node metastases*		
0	1	
1–3	2.74 (0.63, 11.98)	0.181
>4	14.66 (3.24, 66.43)	0.001

Abbreviations: CI=confidence interval; CR=complete response; PR=partial response; SD=stable disease; PD=progression disease.
